# The potential of invasive and non-invasive vagus nerve stimulation to improve verbal memory performance in epilepsy patients

**DOI:** 10.1038/s41598-022-05842-3

**Published:** 2022-02-07

**Authors:** Ann Mertens, Stefanie Gadeyne, Emma Lescrauwaet, Evelien Carrette, Alfred Meurs, Veerle De Herdt, Frank Dewaele, Robrecht Raedt, Marijke Miatton, Paul Boon, Kristl Vonck

**Affiliations:** 1 Department of Neurology, 4BRAIN Research Group, Ghent University Hospital, 9000 Ghent, Belgium; 2 Department of Neurosurgery, Ghent University Hospital, Ghent, Belgium; 3 Department of Electrical Engineering, Eindhoven University of Technology, Eindhoven, The Netherlands

**Keywords:** Neuroscience, Neurology

## Abstract

It has been demonstrated that acute vagus nerve stimulation (VNS) improves word recognition memory in epilepsy patients. Transcutaneous auricular vagus nerve stimulation (taVNS) has gained interest as a non-invasive alternative to improve cognition. In this prospective randomized cross-over study, we investigated the effect of both invasive VNS and taVNS on verbal memory performance in 15 patients with drug-resistant epilepsy. All patients conducted a word recognition memory paradigm in 3 conditions: VNS ON, VNS OFF and taVNS (3-period 3-treatment cross-over study design). For each condition, patients memorized 21 highlighted words from text paragraphs. Afterwards, the intervention was delivered for 30 s. Immediate recall and delayed recognition scores were obtained for each condition. This memory paradigm was repeated after 6 weeks of VNS therapy in 2 conditions: VNS ON and VNS OFF (2-period 2-treatment cross-over study design). Acute VNS and taVNS did not improve verbal memory performance. Immediate recall and delayed recognition scores were significantly improved after 6 weeks of VNS treatment irrespective of the acute intervention. We can conclude that the previously described positive effects of invasive VNS on verbal memory performance could not be replicated with invasive VNS and taVNS. An improved verbal memory performance was seen after 6 weeks of VNS treatment, suggesting that longer and more repetitive stimulation of the vagal pathway is required to modulate verbal memory performance.

*Clinical trial registration number*: NCT05031208.

## Introduction

Cognitive impairment is a common comorbidity in epilepsy, observed in 70–80% of patients^1^. Therefore, apart from improving seizure control, cognitive impairment should be evaluated and addressed in epilepsy patients in order to improve the patient’s overall functioning and quality of life. There is a lack of controlled clinical trials investigating treatments for cognitive impairment in epilepsy patients. The currently available studies investigating drugs or other interventions such as cognitive rehabilitation have shown limited effects^2^. Vagus nerve stimulation (VNS) received CE mark as an adjunctive treatment for drug-resistant epilepsy in 1994 and since then, has become a valuable treatment option in epilepsy centers worldwide^3^. The efficacy and safety of VNS has been studied in multicenter randomized controlled trials and open-label follow-up studies. A reduction in seizure frequency of 50% or more, defining a patient as a responder to the therapy, was demonstrated in at least a third of patients with drug-resistant epilepsy after 1 year of VNS treatment^4,5^. After long-term treatment, efficacy further increased to responder rates above 50%^6^. Although the main outcome parameter in clinical studies was the effect on seizures, improvements in quality of life, mood and cognitive functioning have been reported that did not correlate with a reduction in seizure frequency^7–9^. These findings led to the investigation of VNS as a treatment for drug-resistant depression^10^ (with CE mark in 2001) and cognitive impairment^11^. VNS-induced cognitive modulation has been reported following short-term VNS. In 1999, Clark et al. demonstrated a significantly enhanced word recognition memory in patients with drug-resistant epilepsy recently implanted with VNS, when stimulation was delivered during the consolidation phase of a memory task. Moderate levels of stimulation proved to be most efficient in improving memory performance whereas low or high levels induced no change or even deterioration^12^. This finding was confirmed by the demonstration of deterioration of figural recognition memory with high intensity VNS in the study by Helmstaedter et al.^13^. Ghacibeh et al. found that VNS specifically enhanced the consolidation phase of memory formation, rather than memory retrieval^14^. In contrast to these findings after acute VNS, no convincing evidence of cognitive improvement after long-term VNS has been reported in epilepsy patients^15–17^.

To date, the mechanism of action of VNS and more specifically, the effect on memory performance, has not been elucidated. It has been well established that arousal shortly following a learning event enhances memory consolidation. This process is believed to be related to an interaction between central arousal systems (locus coeruleus (LC), nucleus basalis) and brain regions attributed to affective (e.g. amygdala) and cognitive (e.g. hippocampus) aspects of memory formation mediated by peripheral stress hormones (norepinephrine (NE) and glucorticoïds)^18–21^*.* Preclinical research suggests that the vagus nerve plays a crucial role in this process by transmitting these peripheral neuromodulatory effects to the brain structures involved in memory storage^20,22^. The vagus nerve projects to the nucleus of the solitary tract (NTS) and consequently activates the noradrenergic neurons in the LC. This results in the release of NE in brain structures involved in memory formation such as the hippocampus, the basolateral amygdala and the medial prefrontal cortex^20,23–25^. As this noradrenergic activation has been shown to enhance memory performance, the NTS-LC-NE pathway is believed to play an important role in the memory enhancing effects of VNS^19,26,27^. Apart from NE, other neurotransmitters known to facilitate neural plasticity, such as acetylcholine and serotonin, may be involved in the mechanism of action^28–30^.

More recently, non-invasive neurostimulation modalities have gained interest, aiming to induce a neuromodulatory effect without the need for an invasive procedure. Transcutaneous auricular vagus nerve stimulation (taVNS) is such a non-invasive neurostimulation modality that stimulates the auricular branch of the vagus nerve at the outer part of the ear^31^. Functional brain imaging studies have shown a similar activation pattern with taVNS and invasive VNS, suggesting that taVNS effectively activates the vagal trajectory to the brain and could potentially induce similar effects as invasive VNS^32,33^. Several studies have investigated the effect of taVNS on various aspects of cognition in healthy volunteers: taVNS has been shown to affect post-error slowing^34^, response selection^35,36^, response speed when two action are executed in succession^35^, divergent thinking^37^, inhibitory control processes^38,39^, conflict-triggered cognitive control^40^, flow experience during a task^41^, emotion recognition^42,43^, social information processing^44^ and fear extinction learning^45,46^. The effect of taVNS on memory performance was first investigated by Jacobs et al. in 2015^47^. They found that a single session of taVNS enhanced associative memory performance in older healthy volunteers. More recently, Giraudier et al. found that taVNS improved recollection-based memory performance in healthy volunteers, suggesting that taVNS specifically improves hippocampus-mediated consolidation processes^48^. However, no effect of taVNS was observed on overall memory performance. The latter is in line with a previous study by our group that could not demonstrate an improvement of verbal memory performance after taVNS in healthy volunteers^49^. A possible explanation for these negative findings is the testing of healthy participants. A lower baseline memory performance in epilepsy patients could be more prone to improvement, as indicated by the significant improvement of verbal memory performance in epilepsy patients in the study by Clark et al.^12^. In this prospective randomized cross-over study, we investigated the effect of both invasive VNS and taVNS on verbal memory performance in stimulation-naïve patients with drug-resistant epilepsy during a first session. The effect of invasive VNS on verbal memory performance was re-evaluated after 6 weeks of VNS treatment during a second session. Based on previous research, we hypothesized to find an intensity-dependent effect of acute invasive VNS and taVNS on verbal memory performance. Five research questions were addressed during the statistical analysis with the following hypotheses:

1. We hypothesized that both acute VNS at moderate stimulation intensity and taVNS at pain threshold stimulation intensity would improve verbal memory performance compared to no stimulation at session 1
2. We hypothesized that acute VNS at a higher stimulation intensity would induce less or no improvement in verbal memory performance in session 2
3. We hypothesized that verbal memory performance would be improved after 6 weeks of VNS treatment
4. Similar to invasive VNS, we hypothesized to find an intensity-dependent effect of taVNS on verbal memory performance
5. We hypothesized that the pain experienced during stimulation would significantly differ between interventions

## Methods

### Patients

We included 16 patients with drug-resistant epilepsy who were implanted with VNS at Ghent University Hospital between November 2018 and December 2020. All patients had undergone a presurgical evaluation and were considered unsuitable candidates for epilepsy surgery or refused resective surgery. Patients with an IQ score below 70 were excluded. Written informed consent was obtained from each patient before the beginning of the experimental session. Due to technical problems, the delayed recognition scores of one of the patients could not be obtained. Therefore, this patient was excluded from the analysis, leading to a final sample size of 15 patients. The study protocol was reviewed and approved by the ethics committee of Ghent University Hospital and conformed to the ethical standards of the Declaration of Helsinki.

### Experimental design

**Figure 1:**
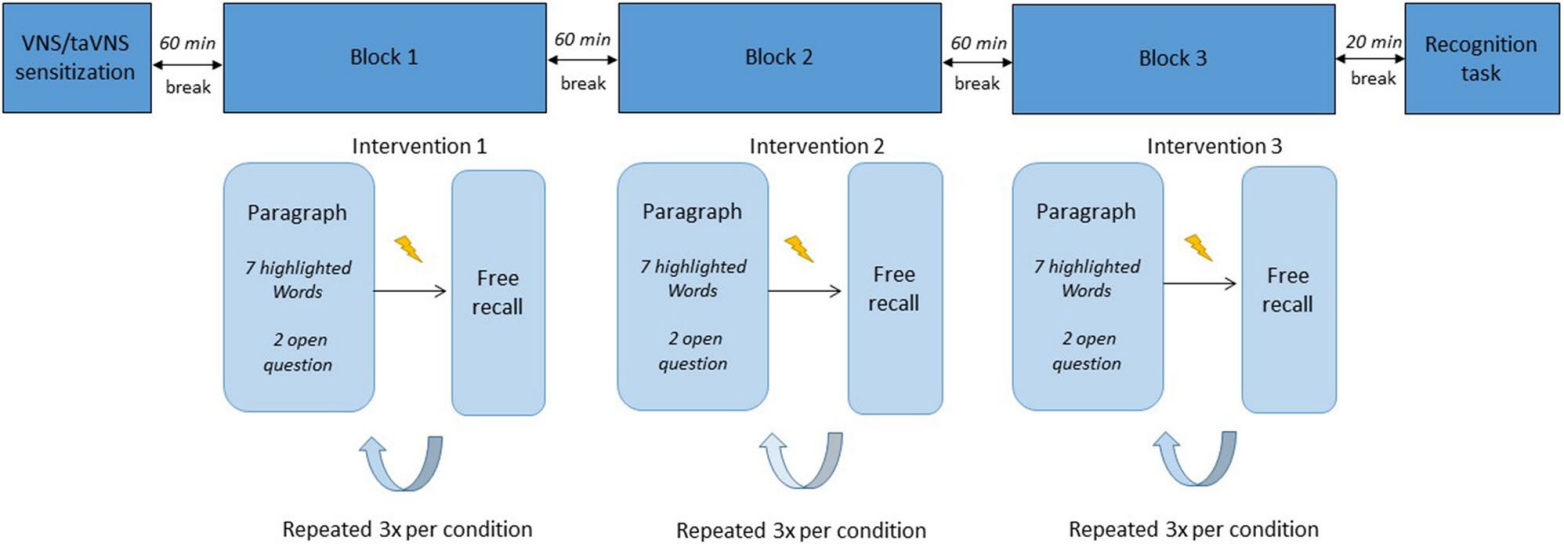
Overview of the first experimental session. After VNS sensitization and programming of the appropriate taVNS settings, a word recognition memory paradigm was conducted in 3 blocks using 3 experimental conditions. All blocks were separated by a 60 min wash-out period. Stimulation () was delivered 2 min after each paragraph and followed by a free recall task. This was repeated 3 times per condition. At the end of the memory paradigm, a final recognition task was performed. The same word recognition memory paradigm was used in the second experimental session consisting of 2 blocks instead of 3.

The first experimental session took place 2 weeks after VNS implantation in stimulation-naïve patients. During this session, patients were admitted to the video-EEG monitoring unit for VNS initiation as part of standard clinical care. A second session was conducted in an ambulatory setting 6 weeks later. All sessions took place in the morning, except for one patient who experienced many absence seizures during the morning of testing. For this patient, both sessions were delayed to the afternoon. During the first session, the VNS device was switched on for the first time. Stimulation was increased to 0.5 mA for 30 s to familiarize the patient with the stimulation sensation. Next, the VNS device was switched off again and the appropriate taVNS stimulation current was chosen according to the pain threshold method. Afterwards, a break of 60 min was implemented to ensure wash-out of stimulation effects before the beginning of the memory task. Each patient conducted the word recognition memory paradigm in 3 conditions: VNS ON, VNS OFF and taVNS (Fig. 1). Therefore, this session had a 3-period 3-treatment cross-over design. A wash-out period of 60 min was implemented between the experimental conditions. After completing the paradigm in all 3 conditions, there was a break of 20 min, followed by a word recognition task. After the experiment, the VNS device was programmed according to standard clinical care and patients were discharged. Six weeks later, this experiment was repeated at session 2. The second session had a 2-period 2-treatment cross-over design as patients conducted the word recognition memory paradigm in 2 conditions: VNS ON and VNS OFF. The same wash-out periods were used in both sessions. During these breaks, patients were asked to perform a relaxing activity. They were not allowed to eat or consume caffeine during the experiment. The investigator was present throughout the experimental sessions to evaluate the occurrence of clinical seizures during testing. In addition, the EEG monitoring during the first session was analyzed to detect (sub)clinical seizures. If seizures with impaired awareness were detected, the session was delayed or the patient was excluded from the statistical analysis. The first session had a duration of approximately 4 h. The second session lasted 2.5 h. The order of the experimental conditions was randomized and balanced across patients. Patients were blinded for the experimental conditions VNS ON and OFF.

### Vagus nerve stimulation

All patients were implanted with the AspireSR® VNS device (LivaNova, Houston, Texas, USA) which consists of 2 helical bipolar stimulating electrodes wound around the left cervical vagus nerve and connected to a subclavicularly implanted pulse generator. The AspireSR® consists of 3 modes of stimulation: a normal mode, a magnet mode and an autostimulation mode. The normal mode provides intermittent stimulation in ON and OFF cycles. Additionally, the magnet mode allows to manually deliver stimulation at the appropriate time by passing a magnet externally across the pulse generator. The AspireSR® is the first VNS device that also contains a seizure detection algorithm that automatically triggers stimulation when an ictal heart rate increase is detected: the autostimulation mode. During the experimental sessions VNS normal mode and autostimulation were set to 0 mA. The magnet mode was programmed to 0.5 mA (VNS ON, session 1), 1 mA (VNS ON, session 2) or 0 mA (VNS OFF). By passing the magnet across the pulse generator, stimulation could be delivered specifically during the consolidation phase of the memory task. Stimulation was delivered for 30 s. At discharge, after the first session, the VNS normal mode was set to 0.5 mA with pulse width of 500 µs, frequency of 30 Hz and a duty cycle of 30 s ON and 5 min OFF, as part of standard clinical care. Magnet and autostimulation mode were programmed 0.25 mA and 0.125 mA higher than normal mode respectively. After 3 weeks, all VNS parameters were increased with 0.25 mA if tolerated during follow-up consultation. Therefore, at session 2, all patients had been treated with VNS for 6 weeks with normal mode stimulation intensities up to 0.75 mA.

### Transcutaneous auricular vagus nerve stimulation

taVNS was delivered by means of the NEMOS® taVNS device (former Cerbomed, Erlangen, Germany). The 2 titan auricular electrodes were placed at the cymba conchae of the left outer ear, a location that is exclusively innervated by the auricular branch of the vagus nerve^50^. Monophasic square pulses were delivered. An alcohol wipe and if necessary electrode spray was used to ensure good electrode contact.The appropriate stimulation current was individually chosen by means of the pain threshold method i.e. taVNS stimulation current was increased in steps of 0.1 mA. Once the patient experienced pain, the stimulation was decreased with 0.1 mA. All patients could perceive the stimulation at the chosen threshold. The frequency was programmed to 25 Hz and pulse width to 250 µs. Stimulation was delivered for 30 s during the consolidation phase of the memory task. Patients were informed about possible side effects of stimulation (e.g. pain, redness of the skin, itching)^51^.

### Word recognition memory paradigm

The memory task in this study was based on the word recognition memory paradigm by Clark et al.^12^ and has previously been reported by our group^49^ (Fig. 1). The paradigm was designed with E-prime software (Psychology Software Tools Inc., Pittsburgh, PA, United States) and conducted on a laptop with a 14-inch screen (Dell, Windows 7). At the beginning of each session, a short introduction was given by the investigator. During the experiment, written instructions were displayed on the screen. Patients were instructed to silently read fragments of text paragraphs. A practice paragraph was given to familiarize the patient with the testing procedures. All paragraphs were chosen from the “wablieft krant,” an online journal in the patients’ native language Dutch, characterized by a low difficulty level. One paragraph was divided into 5 to 6 fragments, displayed separately on the computer screen. Patients could continue to the next fragment by pressing the space bar. In each paragraph, 7 words were highlighted. Patients were instructed to read the paragraph thoroughly and memorize the highlighted words. Immediately afterwards, patients were asked to answer 2 open questions about the content of the paragraph. In accordance with the study by Clark et al.^12^, the intervention (VNS ON/VNS OFF/taVNS) was delivered 2 min after each paragraph in order to stimulate during the consolidation phase of memory formation. After the stimulation, patients were instructed to recall the highlighted words (immediate free recall). Next, patients rated pain during the stimulation using the Wong–Baker FACES Pain Rating Scale ranging from 0 to 10. One text file consisting of 3 paragraphs was used in each experimental condition, leading to a total of 21 highlighted words per condition. During the first session, each patient conducted the paradigm in 3 experimental conditions: VNS ON, VNS OFF and taVNS. At session 2, the word recognition memory paradigm was repeated in 2 experimental conditions: VNS ON and VNS OFF. As each patient performed the paradigm in 5 experimental conditions throughout the study (3 conditions in session 1 and 2 conditions in session 2), 5 different text files were used, each containing 3 distinct paragraphs. Each paragraph was only given once per patient to minimize practice effects. Both the order of text files and conditions were randomized and balanced across patients. At the end of each session, a word recognition task was conducted. During this final task, all highlighted words and an equal number of related and non-related distractor words were individually displayed on the computer screen in a randomized order. The patient was asked to recognize target words (highlighted words) and distinguish them from non-target words (related and non-related words) by pressing a green button when a target word was displayed and pressing a red button for a non-target word. All target and non-target words were controlled for number of characters, frequency and concreteness. The outcome measures of this word recognition memory paradigm were immediate recall score, hit score and discrimination index. The immediate recall score is the percentage of highlighted words that was correctly recalled after each paragraph. The hit score is the percentage of highlighted words that was correctly evaluated as target words during the recognition task. The discrimination index is calculated by subtracting the false alarm score (percentage of related non-target words wrongfully evaluated as target words) from the hit score. The percentage of correctly answered open questions, defined as the attention score, was also taken into account to control for attentional deficits. As the open questions were filled out before administration of the intervention, the attention score should not be affected by the intervention and merely reflects the attentional effort during reading. These outcome measures were calculated for each patient per experimental condition.

### Clinical response after 6 weeks of vagus nerve stimulation

Mean monthly seizure frequency at baseline and after 6 weeks of VNS treatment was calculated based on the patient’s seizure diary before VNS implantation and at experimental session 2. A baseline period of + /− 8 weeks before VNS implantation was used. Patients with at least 50% decrease in mean monthly seizure frequency after 6 weeks of VNS treatment were defined as responders.

### Statistical analysis

Statistical analysis was conducted with SPSS 26 (IBM Corp. Released 2019. IBM SPSS Statistics for Windows, Version 26.0. Armonk, NY: IBM Corp). A linear mixed model analysis was performed with a random intercept for patient. Five research questions were addressed during the statistical analysis:

1. Research question 1: to investigate the effect of the experimental condition on verbal memory performance at session 1 with a 3-period 3-treatment cross-over design, a linear mixed model analysis was performed with immediate recall score, hit score, discrimination index and attention score as the dependent variables and Intervention (VNS ON/VNS OFF/taVNS), order (block1, block2, block3) and their interaction as independent variables.
2. Research question 2: Differences in verbal memory performance at session 2 with a 2-period 2-treatment cross-over design were investigated using a similar analysis with immediate recall score, hit score, discrimination index and attention score as dependent variables and intervention (VNS ON/VNS OFF), order (block1, block2) and their interaction as independent variables.
3. Research question 3: To investigate if memory performance differed between session 1 and session 2 and evaluate a potential relation with the acute intervention and clinical response, a linear mixed model analysis was conducted with immediate recall score, hit score, discrimination index and attention score as the dependent variables and intervention (VNS ON/VNS OFF/taVNS), order (block1,block2,block3), time (session 1, session 2), clinical response (responder/non-responder) and the interactions time*intervention, time*order, time*clinical response, intervention*order, intervention*clinical response and order*clinical response as the independent variables.
4. Research question 4: To explore an association between taVNS stimulation current intensity and memory performance after taVNS, only verbal memory performance scores after taVNS were included in the analysis. The dependent variables were immediate recall score, hit score and discrimination index. The independent variable was taVNS stimulation current.
5. Research question 5: To investigate if pain reporting differed between the interventions, pain score was included as the dependent variable and intervention as the independent variable. This analysis was conducted for session 1 and session 2 separately.

If the interaction effects were not significant, they were excluded to obtain a more simple statistical model. For all statistical tests, the level of statistical significance was set at 0.05. Bonferroni corrections were applied for post-hoc pairwise comparisons to control for multiple comparisons.

## Results

### Patient population

Five male and ten female patients with drug-resistant epilepsy were included in the statistical analysis with a mean age of 39.47 years (standard deviation (SD) 12.61, ranging from 21 to 61 years). All patients had an IQ score > 70 based on the neuropsychological examination during the presurgical evaluation. Seven patients (47.67%) obtained higher education An overview of the demographic and clinical characteristics is reported in Table 1. Medication was not modified between both sessions.

**Table 1:** Overview of demographic and clinical characteristics.

Patient	Age	Sex	IQ	Seizure frequency at baseline	Seizure Frequency After 6 Weeks VNS	Responder	Number of AEDS
1	61	F	83	1.33/month	2/month	No	3
2	24	M	114	+ /- 20/day	+ /- 20/day	No	2
3	36	F	89	12/month	2/month	Yes	2
4	32	M	102	1/month	0/month	Yes	3
5	37	F	92	10/month	10/month	No	3
6	33	F	115	+ /- 8/day	+ /- 2/day	Yes	4
7	21	M	98	0.5/month	0/month	Yes	2
8	52	M	97	+ /- 1- 3/ night	+ /- 0 -4/night	No	4
9	54	F	94	1/day	1/day	No	1
10	41	F	117	+ /- 20/day	+ /- 20/day	No	3
11	51	F	95	0/month	1.5/month	No	2
12	43	M	104	1/month	0.66/month	No	3
13	54	F	75	1/day	0/day	Yes	3
14	28	F	79	2/month	2.5/month	No	4
15	25	F	82	6/month	0/month	Yes	2

AED, anti-epileptic drug; F, female; M, male.

### Verbal memory performance session 1 (Fig. 2)

**Figure 2:**
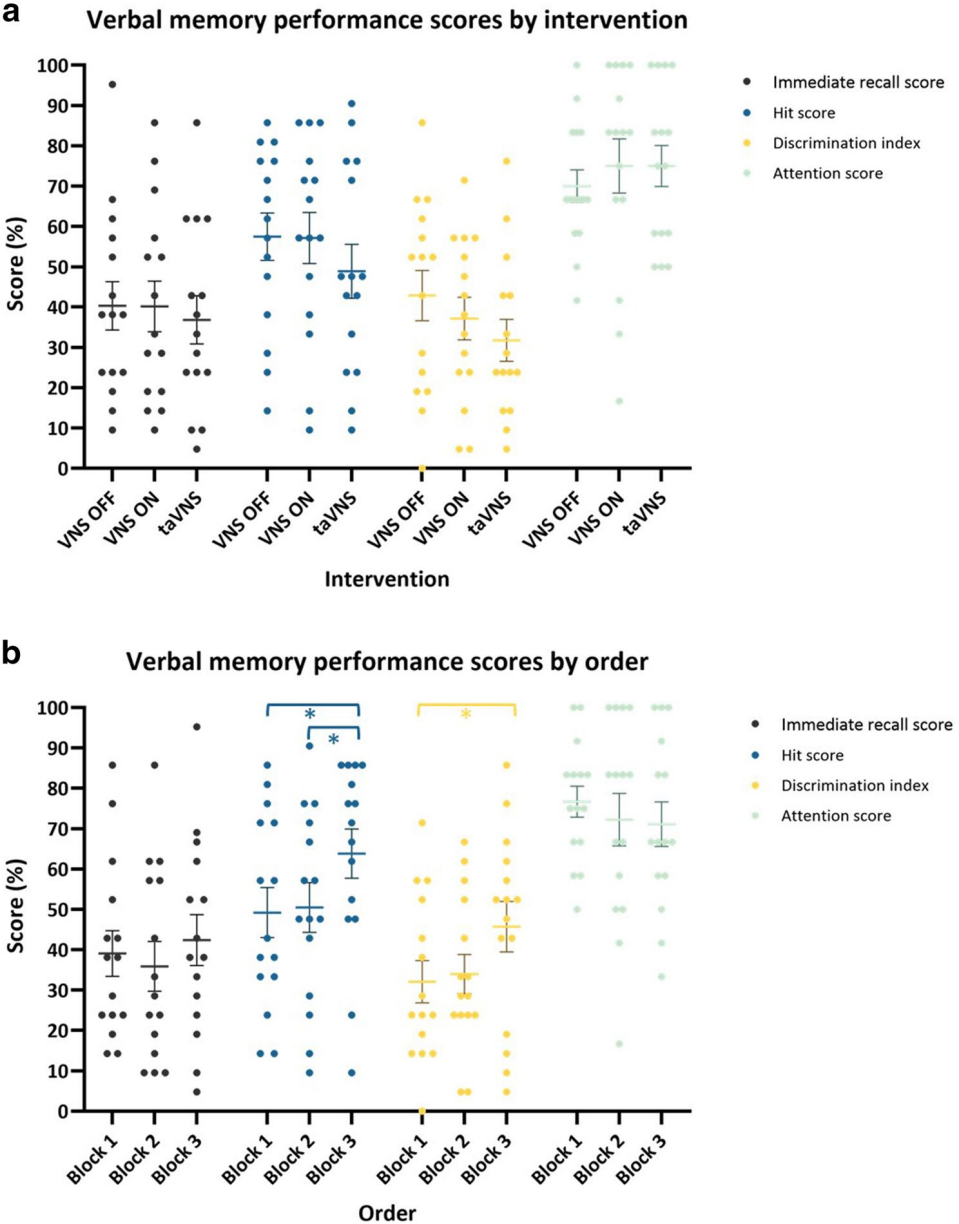
Verbal memory performance by intervention (**a**) and order (**b**) in session 1. (**a**) There was no effect of intervention on all verbal memory performance scores. A trend was seen for lower hit and discrimination index scores after taVNS. (**b**) A significant effect of order was seen for hit scores (*p* = 0.001) and discrimination index (*p* = 0.022).Pairwise comparison demonstrated significantly higher hit scores in block 3 compared to block 2 and block 1 and a significantly higher discrimination index in block 3 compared to block 1. Error bars indicate group mean ± standard error (SE). Significant findings are indicated by an asterix.

An overview of the individual scores on all outcome measures is given in Supplementary Table 1.

#### Immediate recall

The mean immediate recall score was 39.10% (SD 23.05). Linear mixed model analysis showed no significant main effect of intervention (F = 0.36, *p* = 0.700) and order (F = 1.10, *p* = 0.349). The interaction order*intervention was left out of the model as it was not significant.

#### Delayed recognition

- *Hit score*

The mean hit score at session 1 was 54.50% (SD 24.21). Linear mixed model analysis showed no significant effect of intervention (F = 3.22, *p* = 0.056). A trend was seen for lower hit scores after taVNS compared to VNS ON (mean difference 9.44 (95% CI [1.25;17.63]), *p* = 0.026, Bonferroni corrected α = 0.016) with post-hoc pairwise comparisons. There was a significant effect of order (F = 8.56, *p* = 0.001). Pairwise comparison showed a significantly higher hit score at block 3 compared to block 1 (mean difference: 15.34 (95% CI [7.15;23.53]), *p* = 0.001, Bonferroni corrected α = 0.016) and block 2 (mean difference 12.91 (95% CI [4.72;21.10]), *p* = 0.003, Bonferroni corrected α = 0.016). These findings indicate a recency effect where the most recent learned words are better recognized. Since not significant, the interaction order*intervention was left out of the model.

b. *Discrimination index*

The mean discrimination index score at session 1 was 37.25% (SD 21.62). Linear mixed model analysis showed no significant effect of intervention (F = 2.41, *p* = 0.109). Post-hoc pairwise comparison showed a trend for a lower discrimination index after taVNS compared to VNS OFF (mean difference 10,60 (95% CI [0.59;20.60]), *p* = 0.039, Bonferroni corrected α = 0.016). There was a significant effect of order (F = 4.45, *p* = 0.022). Pairwise comparison showed a significantly higher discrimination index at block 3 compared to block 1 (mean difference: 13.81 (95% CI [3.81;23.82]), *p* = 0.009, Bonferroni corrected α = 0.016) and a trend for a higher discrimination index of block 3 compared to block 2 (mean difference 10.77 (95% CI [0.76;20.77]), *p* = 0.036, Bonferroni corrected α = 0.016), indicating again a recency effect. The interaction order*intervention was left out of the model as it was not significant.

#### Attention score

The mean attention score at session 1 was 73.33% (SD 20.54). Linear mixed model analysis showed no main effect of intervention (F = 0.47, *p* = 0.631) and order (F = 0.49, *p* = 0.620). The interaction order*intervention was left out of the model as it was not significant.

#### Pain score

Mean pain score during session 1 was 0.20 (SD 0.43) for VNS OFF, 2.51 (SD 2.98) for VNS ON and 2.62 (SD 2.13) for taVNS. Linear mixed model analysis showed a significant effect of intervention on pain score (F = 6.90, *p* = 0.004). Pairwise comparison demonstrated higher pain scores for VNS ON compared to VNS OFF (mean difference 2.31 (95% CI [0.80–3.82]), *p* = 0.004, Bonferroni corrected α = 0.016) and taVNS compared to VNS OFF (mean difference 2.42 (95% [CI 0.91–3.93]), *p* = 0.003, Bonferroni corrected α = 0.016). No significant difference was demonstrated in pain scores between VNS ON and taVNS.

### Verbal memory performance session 2 (Fig. 3)

**Figure 3:**
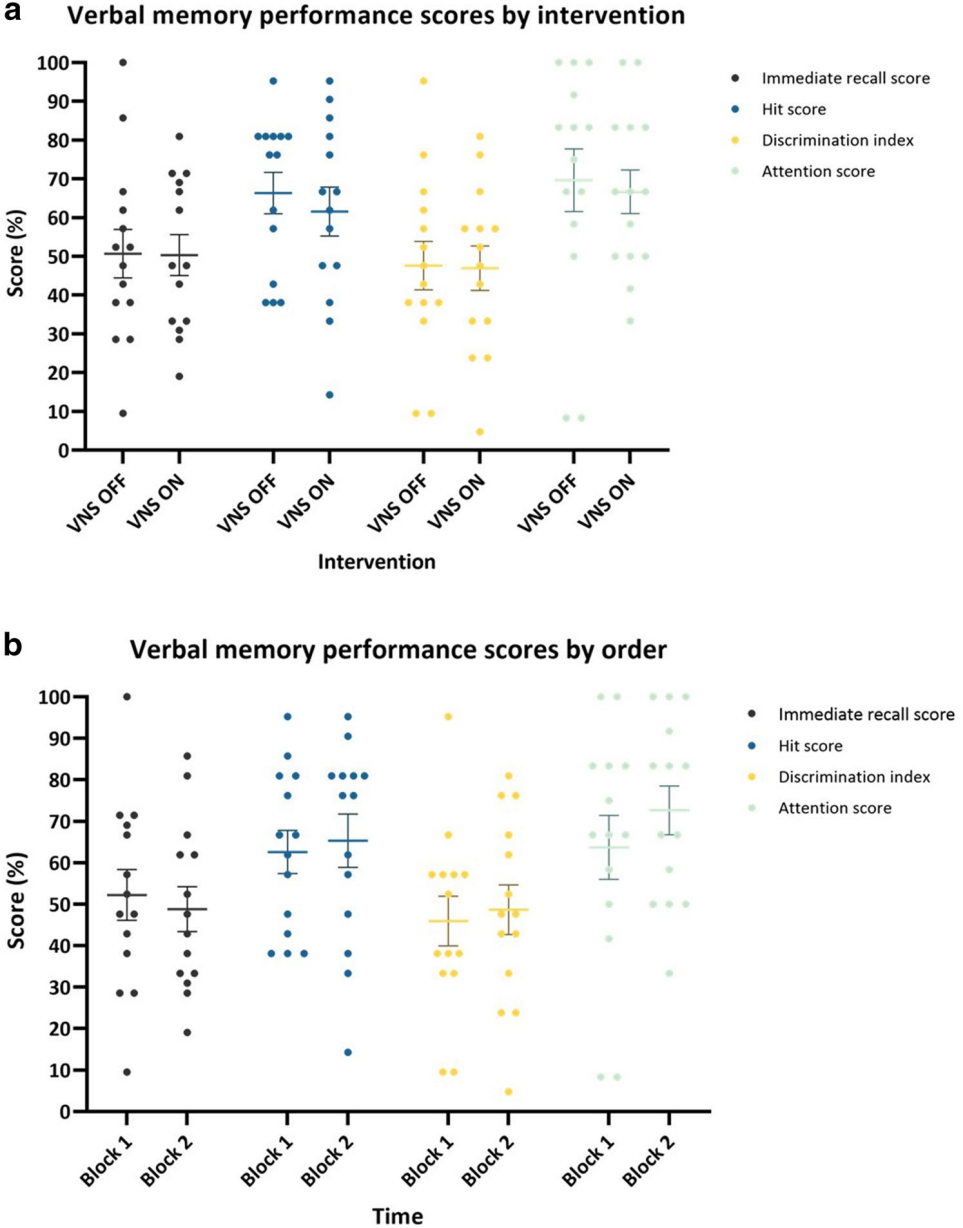
Verbal memory performance by intervention (**a**) and order (**b**) in session 2. There was no effect of intervention or order on all verbal memory performance scores. Error bars indicate group mean ± SE.

The second session of one of the patients was discontinued due to a seizure with post-ictal confusion. Therefore, the memory performance scores of this patient could not be obtained and only 14 patients were included in this analysis. An overview of the individual scores on all outcome measures is given in Supplementary Table 1.

#### Immediate recall

Mean immediate recall score at session 2 was 50.51% (SD 21.26). Linear mixed model analysis showed no main effect of intervention (F = 0.00, *p* = 0.980) and order (F = 0.35, *p* = 0.564). The interaction order*intervention was left out of the model as it was not significant.

#### Delayed recognition

- *Hit score*

Mean hit score at session 2 was 63.95% (SD 21.52). Linear mixed model analysis showed no main effect of intervention (F = 2.43, *p* = 0.145) and order (F = 1.06, *p* = 0.324). Since not significant, the interaction order*intervention was left out of the model.

b. *Discrimination index*

Mean discrimination index at session 2 was 47.28% (SD 22.07). Linear mixed model analysis showed no main effect of intervention (F = 0.07, *p* = 0.798) and order (F = 0.47, *p* = 0.504). The interaction order*intervention was left out of the model as it was not significant.

#### Attention score

Mean attention score at session 2 was 68.15% (SD 25.56). Linear mixed model analysis showed no main effect of intervention (F = 0.52, *p* = 0.483) and order (F = 2.54, *p* = 0.137). Since not significant, the interaction order*intervention was left out of the model.

#### Pain score

Mean pain score during session 2 was 0.00 (SD 0.00) for VNS OFF and 2.88 (SD 2.98) for VNS ON. Linear mixed model analysis showed a significant effect of intervention on pain score (F = 13.08, *p* = 0.003) demonstrating higher pain scores for VNS ON compared to VNS OFF (Mean difference 2.88 (95% CI [1.16–4.60])).

### Difference in verbal memory performance between session 1 and 2 (Fig. 4)

**Figure 4:**
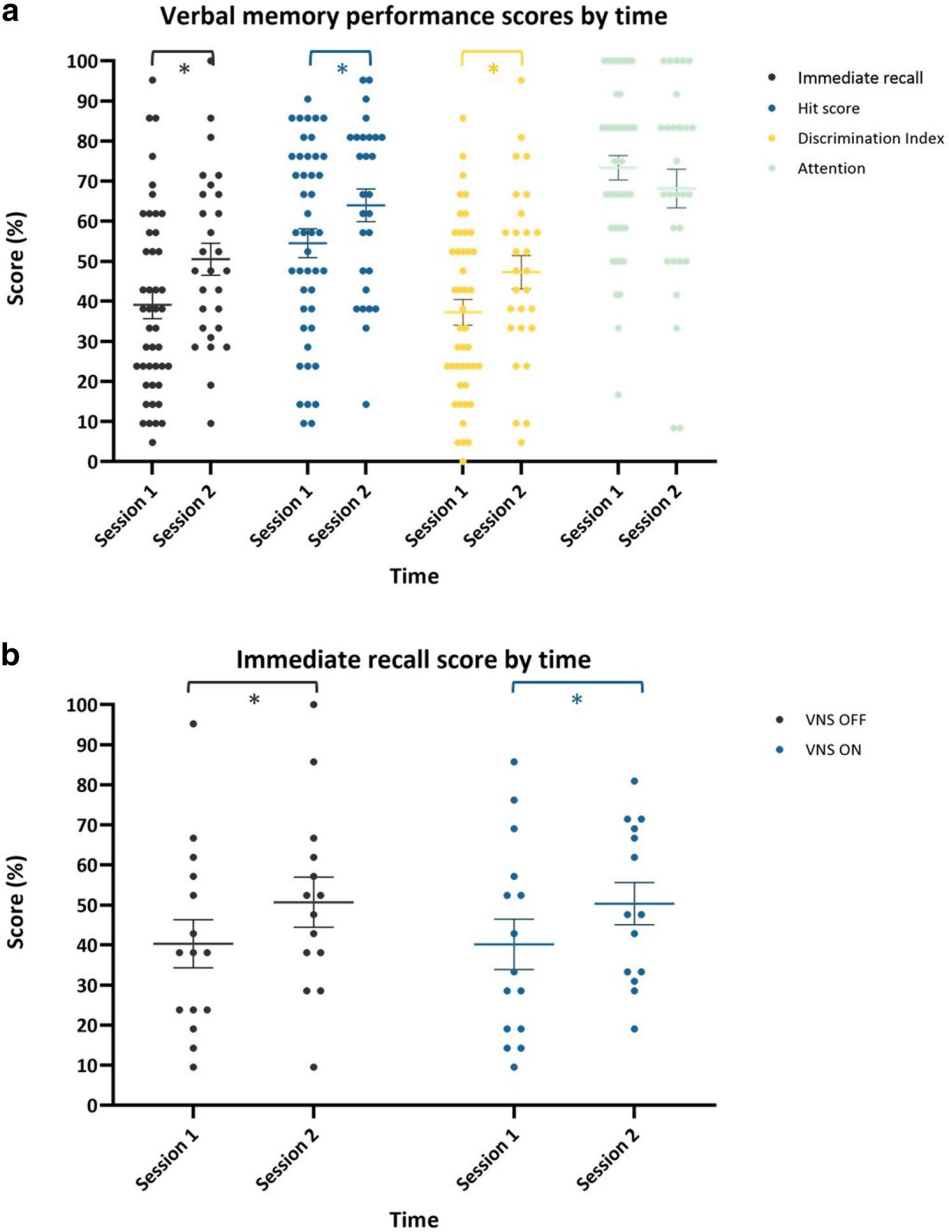
Verbal memory performance by time (**a**) and immediate recall performance by time and intervention (**b**). (**a**) A significant increase in immediate recall score (*p* = 0.001), hit score (*p* = 0.004) and discrimination index (*p* = 0.007) was seen in session 2 compared to session 1. (**b**) No interaction effect of time*intervention was seen, indicating memory performance increased over time irrespective of the experimental conditions, as demonstrated here for immediate recall score. Error bars indicate group mean ± SE. Significant findings are indicated by an asterix.

#### Immediate recall

Linear mixed model analysis showed no significant main effect of intervention (F = 0.34, *p* = 0.715), order (F = 1.32, *p* = 0.277) and clinical response (F = 0.36, *p* = 0.560). A significant main effect of time was found (F = 12.64, *p* = 0.001) demonstrating a higher immediate recall score in session 2 compared to session 1 (mean difference 12.194 (95% CI [5.32;19.07])). All interactions were left out of the model as they were not significant.

#### Delayed recognition

- *Hit score*

Linear mixed model analysis showed no significant main effect of intervention (F = 2.35, *p* = 0.105) and clinical response (F = 0.00, *p* = 0.985). A significant main effect of time was found (F = 8.87, *p* = 0.004) demonstrating a higher hit score in session 2 compared to session 1 (mean difference 11.02 (95% CI [3.60–18.44])). A significant effect of order was also seen (F = 5.97, *p* = 0.005), indicating a recency effect. Since not significant, all interactions were left out of the model.

b. *Discrimination index*

Linear mixed model analysis showed no significant main effect of intervention (F = 1.79, *p* = 0.177) and clinical response (F = 0.02, *p* = 0.892). A significant main effect of time was found (F = 7.77, *p* = 0.007) demonstrating a higher discrimination index in session 2 compared to session 1 (mean difference 12.08 (95% CI [3.39;20.76])). There was also a significant effect of order (F = 3.64, *p* = 0.033), indicating again a recency effect. All interactions were left out of the model as they were not significant.

#### Attention score

Linear mixed model analysis showed no significant main effect of intervention (F = 0.12, *p* = 0.886), time (F = 1.30, *p* = 0.259), order (F = 0.29, *p* = 0.748) and clinical response (F = 0.04, *p* = 0.839). Since not significant, all interactions were left out of the model.

As a trend was seen for lower hit and discrimination index scores after taVNS in session 1 and the taVNS condition was not included in the second session, this may have contributed to the enhancement of verbal memory performance in this analysis. Therefore, the statistical analysis was repeated without the verbal memory performance scores after taVNS. Similar results were found with a significant effect of time for immediate recall score (F = 12,89 *p* = 0.001), hit score (F = 7.36, *p* = 0.010) and discrimination index (F = 6.24, *p* = 0.017).

### Transcutaneous auricular vagus nerve stimulation output current

Mean taVNS stimulation current was 2.42 mA (SD 1.16, range 0.90–5.00). Linear mixed model analysis showed no main effect of taVNS stimulation current on immediate recall score (F = 0.10, *p* = 0.753), hit score (F = 3.01, *p* = 0.106) and discrimination index (F = 1.22, *p* = 0.290).

## Discussion

This is the first study investigating the effect of invasive VNS and taVNS on verbal memory performance in patients with drug-resistant epilepsy in a prospective setting. Immediate recall and delayed recognition scores were not significantly changed by acute VNS or taVNS. We did find an improvement in verbal memory performance after 6 weeks of VNS treatment irrespective of the acute intervention.

In contrast to our hypothesis, immediate recall and delayed recognition scores were not improved by acute invasive VNS and we were therefore unable to demonstrate an intensity-dependent effect of VNS as previously described^12^. No significant differences in attention scores were observed between the interventions, therefore excluding attentional deficits as a reason for these negative findings. Clark et al. demonstrated a significant effect of moderate amplitude (= 0.5 mA) VNS in patients with drug-resistant epilepsy with a similar word recognition memory paradigm^12^. Different inclusion criteria were implemented in the study by Clark et. al. that may explain the contrasting findings. As opposed to the Clark study, many of our patients were treated with benzodiazepines and/or more than 2 anti-epileptic drugs. As polytherapy and benzodiazepines have been found to induce cognitive side-effects^2^, this may have interfered with a potential beneficial effect of VNS. Clark et al. may also have included patients with relatively more severe epilepsy as patients with less than 4 seizures a month were excluded. In contrast, 6 patients in our study reported less than 4 seizures a month during baseline. Only patients with an IQ score below 70 were excluded in our study. Due to the aim of investigating both invasive VNS and taVNS in the first session of our study, the total duration of the experiment was longer (4 h vs 2.5 h in the Clark study) which may have led to a retention interval too long to reliably evaluate memory performance. We did indeed find a significant effect of order in the first session indicating that the most recently memorized words were more easily recognized. Only 2 experimental conditions were investigated in the second session reducing the total duration to 2.5 h. In this shorter session, no recency effect was seen, demonstrating a more reliable evaluation of memory performance at shorter retention intervals. However, as the experimental conditions were counterbalanced across the experimental blocks and no effect of the interaction intervention*order was seen, the longer duration of session 1 alone does not explain our negative findings.

Secondly, we did not find a significant effect of taVNS on verbal memory performance. Moreover, a trend for lower delayed recognition scores was seen after taVNS compared to VNS ON (hit score) and VNS OFF (discrimination index). These findings are in line with the previous study by our group in healthy volunteers, demonstrating that acute taVNS, delivered for 30 s during the consolidation phase of a memory task, did not improve verbal memory performance^49^. We hypothesized the negative findings in the latter study were due to the study population of healthy volunteers who would be less prone to improvement by taVNS compared to epilepsy patients with a lower baseline performance. The current study argues against this hypothesis. Potentially, the duration of stimulation may have been too short to induce significant effects on verbal memory performance. In the study by Jacobs et al., an enhanced associative memory performance was seen after 17 min of continuous taVNS at the tragus^47^. However, as the tragus is not exclusively innervated by the auricular branch of the vagus nerve, this finding is not necessarily related to the activation of the vagal pathway^50^. Giraudier et al. demonstrated an improved recollection-based memory performance after taVNS was delivered in ON/OFF cycles of 30 s before, during and after an encoding task with a total duration of stimulation of 23 min^48^. These longer and/or continuous stimulation protocols may be more effective in modulating verbal memory performance than the short stimulation duration of 30 s implemented in our study. Additionally, only associative and recollection-based memory were affected in the study by Jacobs et al. and Giraudier et al. respectively, indicating that taVNS may affect specific processes of memory formation confined to specific pathways or brain structures instead of inducing a general cognitive improvement.

The taVNS stimulation current was set for each patient individually using the pain threshold method. Therefore, patients were stimulated with different output currents varying from 0.90 to 5.00 mA. As an intensity-dependent modulation of verbal memory performance has been described with VNS, we investigated whether taVNS stimulation current intensity was associated with verbal memory performance. No association was found between stimulation current and verbal memory performance scores. However, stimulation current was compared between subjects whereas a within subject analysis would be the most appropriate method to establish a dose–response relationship. The evaluation of different stimulation currents within one subject was however not feasible in the design of this study.

Thirdly, we demonstrated an improvement in both immediate recall and delayed recognition scores after 6 weeks of VNS treatment. This finding indicates that longer and more repetitive stimulation of the vagus nerve may be required to induce long-term potentiation and effectively modulate memory performance. Only a few studies have investigated the long-term effects of VNS on cognitive functioning in epilepsy patients. Although subjective cognitive improvements have been reported^7,8^, no objective changes in neuropsychological testing were found at 3 to 6 months follow-up^15–17^. This is the first study evaluating verbal memory performance after a short follow-up period during which low stimulation currents were used (0.5–0.75 mA). A longer follow-up would be needed to evaluate if this effect is transient or restricted to moderate stimulation currents, explaining the negative findings in the studies with longer follow-up periods. These results may indicate that the modulatory effect of VNS on memory performance occurs shortly after VNS therapy initiation and is more efficient at moderate stimulation intensities. Of note, due to the relatively short interval between both sessions and the lack of a control condition in this analysis, practice effects may also have contributed to the enhanced performance in the second session and should be taken into account in the interpretation of these findings.

No effect of clinical response on verbal memory performance was seen, indicating that VNS responders did not obtain higher verbal memory performance scores after 6 weeks of VNS compared to non-responders. This suggests that the memory enhancing and seizure suppressing effects of VNS engage different mechanisms and potentially require distinct stimulation protocols. The effect of long-term taVNS on memory performance has not been investigated to this date and should be further explored.

Finally, this study has some limitations. First, the 60-min breaks between the experimental conditions may have been too short to ensure complete wash-out. Studies investigating the enduring effects of VNS on NE have shown inconsistent results ranging from completely transient effects^23^ to elevated NE levels up to 2 h after stimulation^20^. To our knowledge, the enduring effects of taVNS have not been investigated. However, increasing the wash-out periods would have prolonged the duration of the experimental session inducing an even longer retention interval with potential order effects as well as other confounders such as fatigue, diurnal variability, hypoglycemic and post-prandial effects. A second limitation of this study is the incomplete blinding. Patients could easily distinguish the VNS OFF condition from the VNS ON condition, reflected by a significant difference in pain reports after stimulation. In addition, patients were not blinded for the taVNS condition as no sham taVNS condition was included. However, even with incomplete blinding, patients did not perform better after the active stimulation conditions. As a significantly higher pain score was reported after taVNS and VNS ON compared to VNS OFF, the experience of pain during stimulation could potentially decrease memory performance. However, as pain reports were generally low, it seems unlikely this would explain our negative findings. Finally, the finding of an enhanced verbal memory performance after 6 weeks of VNS was not controlled as all patients received active VNS. A randomized controlled trial would provide a higher level of evidence as this would control for placebo effects, practice effects and/or differential experimental circumstances.

## Conclusion

This study is the first prospective study investigating the effect of both invasive VNS and taVNS on verbal memory performance in epilepsy patients. We could not demonstrate an enhanced verbal memory performance after a short stimulus of VNS or taVNS during the consolidation phase of a memory paradigm in this population. These findings are in contrast to previous research, potentially due to the inclusion of a patient population with differential characteristics. This is the first study demonstrating a significant enhancement in verbal memory performance after 6 weeks of VNS treatment. These findings suggest that longer and more repetitive stimulation of the vagal pathway at moderate intensities may be required to effectively modulate memory performance. Future randomized controlled trials including a larger sample size will be needed to validate these results.

## Supplementary Information

Supplementary Information.

## Data Availability

The datasets generated during and/or analysed during the current study are available from the corresponding author on reasonable request.
