# Supplementary material for: The potential of invasive and non-invasive vagus nerve stimulation to improve verbal memory performance in epilepsy patients
Source: Sci Rep. 2022 Feb 7;12:1984. doi: 10.1038/s41598-022-05842-3 (PMC8821667; doi:10.1038/s41598-022-05842-3)
Supplement: Supplementary file 1 — Supplementary Information. [file 41598_2022_5842_MOESM1_ESM.pdf]

The potential of invasive and non-invasive vagus nerve stimulation to improve verbal memory performance in epilepsy patients

Ann Mertens[1*], Stefanie Gadeyne[1], Emma Lescrauwaet[1], Evelien Carrette[1], Alfred Meurs[1], Veerle De Herdt[1], Frank Dewaele[2], Robrecht Raedt[1], Marijke Miatton[1], Paul Boon[1,3] , Kristl Vonck[1]

[1]Department of Neurology, 4BRAIN research group, Ghent University Hospital, Ghent, Belgium

[2]Department of Neurosurgery, Ghent University Hospital, Ghent, Belgium

[3]Department of Electrical Engineering, Eindhoven University of Technology, Eindhoven, The Netherlands

Supplementary table 1: overview of individual scores

Session 1:

**Patient**	**Order**	**Intervention**	**Immediate recall score (%)**	**Hit score (%)**	**Discrimination index (%)**	**Attention score (%)**
**1**	Block 1	VNS ON	28,57	71,43	23,81	100,00
	Block 2	VNS OFF	9,52	66,67	52,38	50,00
	Block 3	taVNS	33,33	76,19	52,38	50,00
**2**	Block 1	VNS ON	52,38	33,33	28,57	83,33
	Block 2	taVNS	23,81	23,81	23,81	100,00
	Block 3	VNS OFF	66,67	61,90	57,14	83,33
**3**	Block 1	VNS ON	42,86	57,14	57,14	83,33
	Block 2	taVNS	42,86	47,62	42,86	83,33
	Block 3	VNS OFF	38,10	52,38	52,38	66,67
**4**	Block 1	VNS ON	76,19	38,10	38,10	75,00
	Block 2	taVNS	85,71	42,86	28,57	100,00
	Block 3	VNS OFF	95,24	85,71	85,71	91,67
**5**	Block 1	VNS OFF	23,81	14,29	14,29	66,67
	Block 2	VNS ON	14,29	9,52	4,76	66,67
	Block 3	taVNS	4,76	9,52	9,52	100,00
**6**	Block 1	VNS ON	85,71	85,71	71,43	91,67
	Block 2	VNS OFF	61,90	76,19	66,67	83,33
	Block 3	taVNS	61,90	76,19	76,19	83,33
**7**	Block 1	VNS OFF	23,81	80,95	19,05	66,67
	Block 2	taVNS	9,52	71,43	23,81	50,00
	Block 3	VNS ON	28,57	85,71	14,29	33,33
**8**	Block 1	taVNS	61,90	33,33	14,29	50,00
	Block 2	VNS ON	57,14	57,14	23,81	66,67
	Block 3	VNS OFF	42,86	80,95	61,90	66,67
**9**	Block 1	taVNS	38,10	14,29	14,29	100,00
	Block 2	VNS OFF	57,14	28,57	23,81	100,00
	Block 3	VNS ON	52,38	47,62	42,86	100,00
**10**	Block 1	VNS OFF	38,10	57,14	42,86	83,33
	Block 2	taVNS	61,90	90,48	61,90	83,33
	Block 3	VNS ON	69,05	85,71	52,38	100,00
**11**	Block 1	VNS OFF	19,05	76,19	52,38	58,33
	Block 2	VNS ON	19,05	76,19	57,14	83,33
	Block 3	taVNS	23,81	85,71	42,86	58,33
**12**	Block 1	taVNS	23,81	42,86	23,81	75,00
	Block 2	VNS OFF	23,81	47,62	28,57	41,67
	Block 3	VNS ON	19,05	66,67	47,62	41,67
**13**	Block 1	VNS OFF	14,29	38,10	,00	58,33
	Block 2	VNS ON	9,52	57,14	33,33	16,67
	Block 3	taVNS	9,52	47,62	4,76	58,33
**14**	Block 1	VNS ON	14,29	71,43	57,14	83,33
	Block 2	taVNS	28,57	47,62	33,33	58,33
	Block 3	VNS OFF	52,38	71,43	66,67	66,67
**15**	Block 1	taVNS	42,86	23,81	23,81	75,00
	Block 2	VNS ON	33,33	14,29	4,76	100,00
	Block 3	VNS OFF	38,10	23,81	19,05	66,67

Session 2:

Patient	Order	Intervention	Immediate recall score (%)	Hit score (%)	Discrimination index (%)	Attention score (%)
**1**	Block 1	VNS OFF	28,57	76,19	9,52	66,67
	Block 2	VNS ON	33,33	76,19	23,81	50,00
**2**	Block 1	VNS ON	42,86	57,14	57,14	83,33
	Block 2	VNS OFF	85,71	76,19	76,19	83,33
**3**	Block 1	VNS ON	/	/	/	/
	Block 2	VNS OFF	/	/	/	/
**4**	Block 1	VNS OFF	100,00	95,24	95,24	100,00
	Block 2	VNS ON	80,95	80,95	80,95	100,00
**5**	Block 1	VNS OFF	28,57	38,10	38,10	75,00
	Block 2	VNS ON	19,05	14,29	4,76	50,00
**6**	Block 1	VNS ON	69,05	66,67	57,14	83,33
	Block 2	VNS OFF	66,67	57,14	47,62	100,00
**7**	Block 1	VNS ON	47,62	85,71	52,38	66,67
	Block 2	VNS OFF	38,10	80,95	52,38	91,67
**8**	Block 1	VNS ON	66,67	47,62	33,33	41,67
	Block 2	VNS OFF	42,86	61,90	42,86	58,33
**9**	Block 1	VNS ON	71,43	61,90	57,14	100,00
	Block 2	VNS OFF	52,38	80,95	66,67	100,00
**10**	Block 1	VNS ON	71,43	66,67	66,67	58,33
	Block 2	VNS OFF	61,90	80,95	61,90	83,33
**11**	Block 1	VNS OFF	38,10	80,95	57,14	66,67
	Block 2	VNS ON	61,90	95,24	76,19	50,00
**12**	Block 1	VNS OFF	57,14	42,86	33,33	8,33
	Block 2	VNS ON	30,95	47,62	47,62	66,67
**13**	Block 1	VNS OFF	9,52	38,10	9,52	8,33
	Block 2	VNS ON	33,33	33,33	23,81	33,33
**14**	Block 1	VNS OFF	52,38	80,95	38,10	50,00
	Block 2	VNS ON	47,62	90,48	42,86	66,67
**15**	Block 1	VNS OFF	47,62	38,10	38,10	83,33
	Block 2	VNS ON	28,57	38,10	33,33	83,33
